# Every Patent Foramen Ovale Should Be Closed

**DOI:** 10.3390/jcm13113355

**Published:** 2024-06-06

**Authors:** Bernhard Meier

**Affiliations:** Department of Cardiology, University of Bern, 3012 Bern, Switzerland; bernhard.meier@gmx.net; Tel.: +41-79-300-2676

**Keywords:** angina pectoris, atrial septal defect, collateral benefit, device closure, left atrial appendage, mechanical vaccination, migraine, myocardial infarction, patent foramen ovale, paradoxical embolism, peripheral embolism, primary prevention, stroke, transient ischemic attack

## Abstract

At present, the patent foramen ovale (PFO) does not receive the deserved medical attention. The PFO poses a serious threat to health and even the life of mankind. The first respective case report in the medical literature dates back to the 19th century. It led to death. The fact that a PFO is present in roughly 25% of people underscores its overall potential to cause harm. Yet at the same time, the sheer number discourages the medical community from screening for it and from treating it. About 5% of the population have particularly dangerous forms of PFOs. Such PFOs portray a high enough risk for clinical events, the likes of death, stroke, myocardial infarction, or ocular, visceral, and peripheral embolism, to justify screening for them. Highly significant health incidents being at stake, it appears obvious that PFO closure should be used for primary prevention. This is supported by the fact that closing a PFO is the simplest intervention in cardiology, with presumably the highest clinical yield. Being mainly a preventive measure, PFO closure represents a mechanical vaccination. When closing PFOs for one of the rarer therapeutic indications (migraine, platypnea orthodeoxia, etc.), patients automatically profit from the collateral benefit of getting, at the same time, mechanically vaccinated for life against paradoxical embolism. Vice versa, closing a PFO for the prevention of paradoxical embolism betters or cures migraine or exercise dyspnea not infrequently, thereby improving quality of life as a collateral benefit.

## 1. Introduction

The closure of a patent foramen ovale (PFO) with a percutaneous occluder device has been around for over 30 years [1]. A dedicated device for the PFO, the Amplatzer PFO Occluder, was first used on 10 September 1997 [2]. As practically the only device in interventional cardiology, it has not needed, and therefore not seen, any significant iteration since its first-in-man use. To date, the Amplatzer PFO Occluder has gathered an impressive record to have worked virtually flawlessly in hundreds of thousands, if not millions, of patients. It comes as no surprise that the device has begot a fair number of duplicates and derivatives.

## 2. PFO Closure Procedure

Closing a PFO with an Amplatzer occluder or a comparable device is extremely simple, innocuous, and effective. After a short and steep learning curve, it can be accomplished by any invasive cardiologist within less than 30 min. There is no necessity for sedation of patients. In fact, there is no reason for them not to leave the catheterization laboratory on foot holding the venous femoral puncture like one holds the elbow after donating blood. There are no physical restrictions after such a PFO closure and even sports including underwater diving are possible on the same day. Medical treatment after the procedure can be stripped down to 100 mg of acetylsalicylic acid daily for a month or two without a preloading or loading dose. Later problems to be looked for are so rare that no further checkups are medically required. Nonetheless, for ruling out a clinically significant residual shunt (to be expected in less than 10% of cases), it is recommendable to perform a transthoracic contrast echocardiogram (TTE) with a bubble test at the end of a sustained Valsalva maneuver after 3 to 6 months. To also reliably uncover small residual shunts (to be expected in about 20% of cases, but clinically irrelevant in most instances) or significant thrombi on the device (a risk of less than 1 in 500), a contrast transesophageal echocardiogram (TEE) is required. Most physicians and patients want to make sure that everything is perfect, and follow-up contrast TTEs or TEEs are standard in the majority of institutions.

The intervention in its economical and frugal version is a same-day procedure, guided solely by fluoroscopy. The patient usually arrives at the catheterization laboratory with a venous line, having received some prophylactic intravenous or oral antibiotics. The intervention starts with a local anesthesia at the right groin, a small skin incision, and an intravenous bolus of 5000 units of heparin.

The femoral vein is punctured, and a conventional 0.035″ guidewire with a U-tip is inserted through the puncture needle. In nearly half of cases, the guidewire spontaneously passes the PFO into the left atrium when advanced up from the inferior vena cava through the right atrium. If not, it pays out to recross the right atrium a couple of times, because the wire may not pass at first but rather at one of the following attempts. The initial attempt to pass the atrial septum with a U-tip wire tries to take advantage of the fact that the U-tip wire can only pass through the slit-like PFO. A straight wire or a catheter may inadvertently pass through a tiny atrial septal defect (ASD) in the septum primum. Small cribriform ASDs often go unnoticed in prior echocardiograms.

If the wire fails to pass, a 4 to 6 French (F) multipurpose catheter is inserted over the wire without a sheath. It may be necessary to actually screw the catheter through the skin and any tense subcutaneous tissue to be able to enter the vein over a regular guidewire. The multipurpose catheter is then parked at the level of the diaphragm pointing to the left shoulder, i.e., to 2 o’clock, in a frontal view. The wire is advanced several times deep into the right atrium until it arrives at the 2 o’clock position. This usually means that the left atrium is reached. In the frontal view, a similar wire trace may appear with an inadvertent route through the right ventricle into the left pulmonary artery. This path, however, almost invariably causes ventricular ectopy during wire advancement. After checking the wire position in a left anterior oblique or lateral view, the erroneous passage through the right ventricle into a pulmonary artery is identified by a bow of the wire on the left side of the screen close to the sternum. The correct route into the left atrium through the PFO shows the wire straight on the right side of the screen in that view.

**Figure 1:**
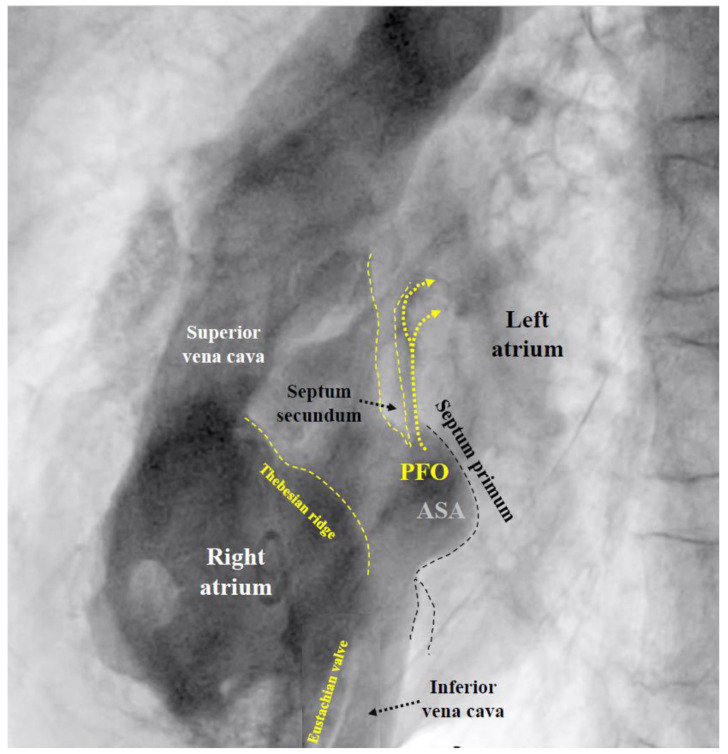
**Fluoroscopic contrast imaging of a patent foramen ovale (PFO)** (left anterior oblique view). All important features are visible, including the Eustachian valve and the Thebesian ridge, a ledge in the free wall of the right atrium, confoundable with the atrial septum. ASA = atrial septal aneurysm.

If the PFO cannot be passed despite changing the direction and position of the multipurpose catheter, the U-tip wire may be removed and the tip straightened by scratching the back of the curve with the thumb nail. Should the now straight wire still not pass, the PFO is searched for with the tip of the multipurpose catheter. If even that fails, a right atriogram with contrast medium is performed to understand the anatomy (Figure 1).

Once the PFO is crossed, the guidewire is kept wherever it spontaneously wound up, be that the left atrium itself, the left atrial appendage (LAA), or a pulmonary vein. In the cases it was used, the multipurpose catheter is now removed. In tall patients, a regular-length guidewire may be too short to manually keep the guidewire tip in the left atrium while exiting the multipurpose catheter. A small caliber syringe or a power injector can be connected to the multipurpose catheter once the distal tip of the wire has vanished into the proximal end of the catheter to forcefully inject saline or contrast medium while the multipurpose catheter is fully withdrawn. This keeps the wire still in the left atrium. Particularly if a second person injects or a power injector is used, the tip of the multipurpose catheter has to be manually stopped by the operator as soon as it exits the skin to avoid uncontrolled ejection of the catheter accompanied by a shower to the environment with the injected fluid.

As soon as the multipurpose catheter is removed, the puncture site is inspected for exclusively venous bleeding. If there is arterial bleeding, a new puncture will be required. It has to be assumed that the femoral vein was inadvertently entered by traversing an artery. For obvious reasons, it must be avoided to insert the 8 to 10 F sheath required for device delivery over a wire passing through an artery into the femoral vein. In the mentioned frequent cases, the wire had passed directly through the PFO without using a multipurpose catheter; it is equally important to check the access path into the femoral vein before fully inserting the delivery sheath. This is achieved by advancing only the tapered tip of the sheath and then withdrawing it briefly to ascertain venous-only bleeding.

The sheath required for device implantation can be inserted over the regular guidewire in place. For passing through the skin into the femoral vein, a screwing rotation is recommended to avoid wire kinking. After that, the path is straight and no more resistance will occur that would require additional stiffness of the guidewire.

Once in the left atrium, the obturator (thick-wall inner part) of the sheath is removed and backflow of arterial blood from the left atrium is ascertained, with the outer end of the sheath held at or below atrial level at the side of the patient. This is where the sheath should be kept and handled throughout the entire case.

**Figure 2:**
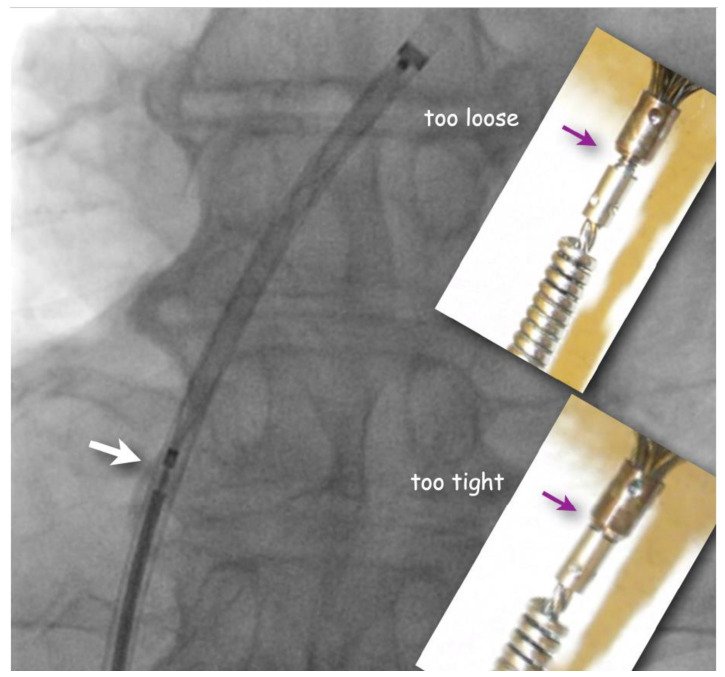
**Fluoroscopic check of correct attachment of Amplatzer(-like) occluders** (frontal view). A small gap (white arrow) between pusher screw collar and screw nut on the device guarantees that the occluder is still safely attached but will be easily detachable once positioned. The inserts show screw positions that should be corrected by turning the pusher cable either clockwise (if too loose) or counterclockwise (if too tight) before advancing the occluder out of the sheath.

The flushed short loader sheath containing the selected preloaded occluder with its tip just peeking out is connected to the backbleeding sheath and the device is advanced to the distal tip of the sheath without any simultaneous flushing. Before exiting the sheath, it is made sure that the screw used for fixing the original Amplatzer device and most of its derivatives during delivery is still properly tightened, but not too much. A small gap ought to be visible (Figure 2). If there is no gap, one or two full left (counterclockwise) turns are recommended, during which an unblocking click is usually felt. This assures that the screw is not tightened overly fast so that the occluder would swivel with the loader cable rather than being released when the unscrewing is attempted at the end of occluder positioning. Too generous a gap results from inadvertent partial unscrewing of the occluder on the way up through the sheath. That needs to be corrected with turning right (clockwise) on the loading cable. In the extremely rare case that the screw is seen to be completely disconnected, the device is not implanted but pulled out of the sheath with a snare and reattached properly before reinsertion and implantation.

The remaining steps are carried out in a left anterior oblique fluoroscopic projection. The left disc is pushed out of the sheath. It may not be clearly visible at first, but the waist of the device is now shown at the tip of the sheath. The system is then pulled back as a unit. As soon as the left disc aligns with the atrial septum, it will infallibly become visible in its profile in the left anterior oblique projection. Now, the right disc is released by keeping the left disc snugly cuddled to the septum and by withdrawing the sheath until proximal to the screw. At this moment, everything is relaxed (no more pulling on sheath or device) and the right disc will automatically align to the right side of the septum or it can be pushed there.

**Figure 3:**
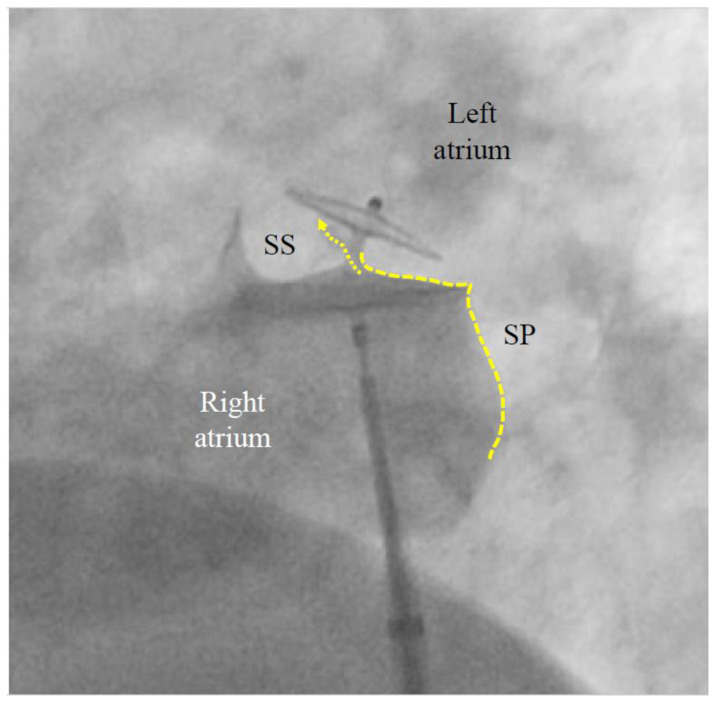
**Contrast medium injection before release of a patent foramen ovale (PFO) occluder** (left anterior oblique view). The left (cranial) part of the 25/18 mm Amplatzer PFO Occluder correctly bites into the septum secundum (SS). This is called a positive Pacman sign and is required for safe release. The original channel of the PFO is now blocked (dotted arrow). The septum primum (SP) is indicated with a dotted line. Its part caudal to the occluder is ideal for possibly later required fluoroscopy-guided transseptal punctures. A pre-release situation like this will usually have the device swivel up to a clockwise quarter turn upon release. The final occluder position is expected to be more vertical and parallel to the atrial septum with no more dent in the SP.

A contrast medium injection is carried out after flushing the sheath and after adjusting the projection so that both discs are seen in perfect profile without any overlap. It will show all necessary details (Figure 3). The device is then released, i.e., unscrewed from the pusher cable or liberated by whatever attachment and release mechanism is used with alternative occluders. A further contrast medium injection after readjusting the projection is required only if the device moves significantly upon release. This typically happens in the presence of an atrial septal aneurysm (ASA) or due to significant position distortion by the sheath or pusher gear before release (Figure 3).

The patients can comfortably and safely hold the groin after having been helped to place their fingers in the right position before or after withdrawing the sheath. In a supine position, very little pressure is required to prevent bleeding by withholding venous pressure of less than 10 mmHg. A hematoma cannot form with such a low pressure. Some operators close the skin nick with a figure-of-eight suture. Once stitches or a finger block the only possible blood exit, the one through the skin, the femoral vein can no longer shed blood. When standing up, the venous pressure abruptly increases to the weight of the column of blood extending from the groin to the tip of the head. This corresponds to about 60 mmHg, requires a more robust manual compression (a just-applied figure-of-eight suture is no longer sufficient), and also carries the risk of a hematoma or copious bleeding to the outside through an unstitched skin nick.

**Figure 4:**
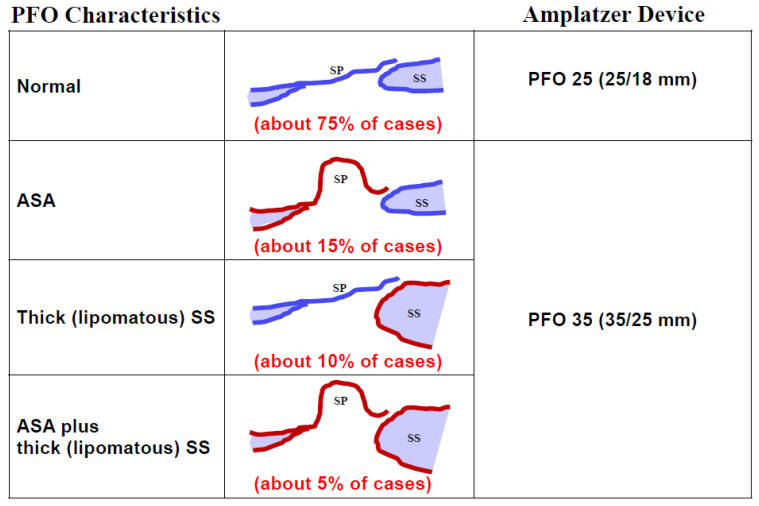
**Device size selection for closure of a patent foramen ovale (PFO) made simple.** The length of the tunnel is not relevant. ASA = atrial septal aneurysm; SP = septum primum; SS = septum secundum.

Regarding occluder size, practically all PFOs can be fit with either a 25 mm PFO occluder (right disc 25 mm and left disc 18 mm) or a 35 mm PFO occluder (35 mm, 25 mm), when using Amplatzer occluders (Figure 4). The 35 mm occluder will be required in about 25% of cases, those with either a marked frailty and mobility of the septum primum (generally referred to as ASA), a lipomatous (thick triangular) septum secundum, or both. The production and use of occluders with a smaller left disc than the right disc make sense. The flap-valve-like PFO will be pulled shut even by a small left disc. A smaller disc on the left side reduces overall contact with the pulsating aorta, the most common cause of the rare but ominous erosion of a free atrial wall by a PFO occluder. The right disc must be large enough to avoid disengagement from the septum secundum. This problem is also called unhinging from the Pacman position and is the consequence of too small a right disc in the presence of an ASA, a lipomatous septum secundum, or both [3].

## 3. When and How to Screen for a PFO

Venous thromboembolism, the prerequisite for a PFO to cause harm, is hardly an issue until late adolescence but its incidence exponentially increases with older age [4,5] and with morbidity. Oral contraception represents an early potential harbinger of paradoxical embolism in the presence of a PFO. Migraine in young people constitutes an attractive indication for PFO screening. PFO closure for migraine has a good chance for symptomatic relief [6] and offers, as a collateral bonus, lifelong primary prevention—mechanical vaccination [7]—against later paradoxical embolism.

**Figure 5:**
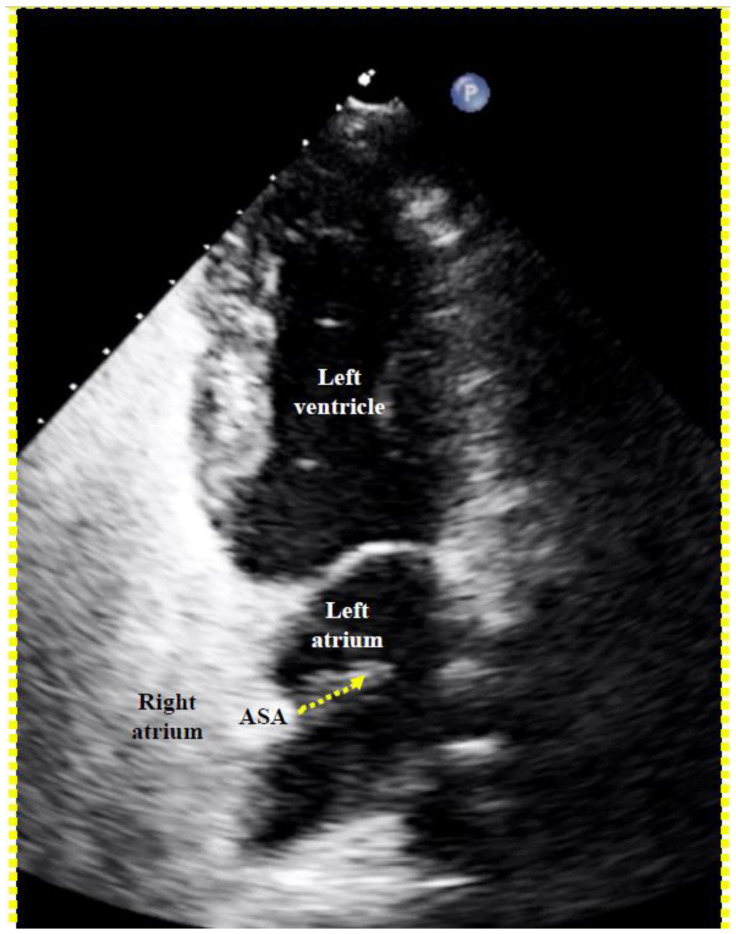
**Proof of a patent foramen ovale (PFO) with right-to-left shunt with transthoracic echocardiography.** A right-to-left bubble shunt (dotted arrow) is demonstrated with its origin in the region of a conspicuous atrial septal aneurysm (ASA).

To screen for dangerous PFOs, i.e., large ones or those associated with an ASA [8], a Eustachian valve [9,10,11,12], or a Chiari network [13], a bubble test during a TTE suffices (Figure 5). The contrast liquid has to be injected at the end of a sustained Valsalva maneuver. A Valsalva maneuver, or pressing into the upper belly by a medical person during TEE if a sedated patient cannot perform a Valsalva maneuver, blocks the backflow of the venous blood into the thorax. Both atria become volume-depleted within 10 to 20 s. Upon Valsalva or pressing release, the venous blood gushes into the right atrium first, thereby pushing the PFO open towards the still underfilled left atrium.

The venous inflow into the right atrium from the inferior vena cava is directed onto the PFO, even more so in the presence of a Eustachian valve [9,10,11,12] or a Chiari network [13]. The inflow of the superior vena cava is not. Hence, injecting the contrast medium in a leg vein would be ideal [14,15]. Yet this is impractical, and under most circumstances, an arm vein has to do. A state-of-the-art bubble test during a TEE or a transcranial Doppler examination [16] in addition detects small PFOs that harbour a smaller (albeit still relevant) risk for paradoxical embolism.

Ear oximetry represents an interesting PFO screening technique first published over 20 years ago [17]. Regrettably, it has not been further pursued since 2013 [18]. It is the only screening method that could be applied in school classrooms or shopping malls. The proband performs a sustained Valsalva maneuver with a graphical oximeter attached to an ear lobe. At the Valsalva release, when any large PFO will briefly open, a small tsunami of venous blood will run through the PFO to reach the ear lobe. The oxygen saturation curve dips momentarily. The depth and width of the dip correspond to the importance of the PFO [18].

Obviously, even with such a simple test, medical logistics and funding are lacking to screen the world population. Having said that, every situation even remotely reminiscent of a PFO problem should be considered a reason and opportunity for a screening test. In the realm of neurology that pertains to migraine, particularly but not exclusively if associated with aura, and to any cerebral event (not only to those called cryptogenic in young and otherwise healthy patients). The PFO causes more paradoxical embolism in old and sick people because of their propensity for venous thromboembolism, the prerequisite for paradoxical embolism. The absolute respective risk for PFO carriers increases geometrically with age and disease. The relative risk decreases due to the aggregated hazard of accumulating competitive causes for systemic arterial occlusions. In the realm of cardiology, one should screen for a PFO and occlude it if found with every myocardial infarction (MI) that looks in any way embolic. Concomitant atherosclerosis by no means acquits the PFO. It rather heightens its risk to cause an MI. The prevalence of both atherosclerosis and venous thrombosis grows in unison with age and comorbidities. Cardiologists should suspect—based on the blood volume distribution in the systemic circulation—one PFO-associated MI per every three PFO-associated cerebral events identified by neurologists. We can add to this that PFO-associated cerebral events are grossly underdiagnosed as they are still only looked for in young and otherwise fit patients, notably the patient group with the lowest absolute respective risk.

Neglected screening for a PFO is only one of the reasons for underused closure of PFOs. Another reason is the false assumption that first PFO-related events be benign and leave time for reaction. PFO case number one in the medical literature was fatal [19]. PFO carriers as a percentage of the population decrease with age [20,21], pointing to selective mortality of people with a PFO. Moreover, in a propensity-matched comparison among patients with a PFO and an index event, mortality was significantly lower over ten years of follow-up in the about 150 subjects with PFO closure than in the same number with purely medical treatment [22]. 

PFOs direly need to receive the medical attention they deserve. PFO closure effectively addresses potentially life-threatening problems with an intervention comparable, in intricacy, to mending teeth. Completely normal life resumes as soon as a couple of hours after the act. The technical success rate is virtually 100%. The long-term clinical efficacy is over 90%. Significant late problems are exotically rare. Actual costs of PFO closure amount to less than EUR 10,000.

Irrespective of the presence of established causes of cerebral events, such as atherosclerosis or atrial fibrillation (AF), the PFO must be looked for. It is a potential culprit for any such accident—the easiest to diagnose and to eliminate, for that matter. Interestingly, the term cryptogenic strokes initially encompassed patients with AF. Only later, AF was promoted to an established cause of stroke. That made sense, but it makes equal sense and is overdue to classify a PFO as a non-cryptogenic stroke cause and also remove it from the basket of embolic stroke of undetermined source called ESUS [23]. Table 1 proposes an adapted classification of embolic events. The potential to cause an embolic event of paroxysmal AF with rare and short episodes is much smaller than that of a high-risk PFO. In both cases, the stroke cause is usually assumed and not proved, with rare exceptions [24,25]. The argument that AF requires oral anticoagulation (OAC) for life, which, at the same time, protects against PFO-mediated events, can be rebutted in two ways. First, OAC provides only limited protection due to its notoriously poor compliance and required pauses. Second, easily combinable PFO and LAA closure (adding only a few minutes and the cost of a second occluder) [26] obviates the need for OAC for AF with its incessantly accruing bleeding risk, exponentially climbing with age. The term mechanical vaccination applies to PFO [7] and LAA [27] closure and both are oblivious to any compliance issue.

## 4. Compelling and Established Indications for PFO Closure (Table 2)

At present, randomized data regarding PFO closure for ischemic events are limited to patients under the age of 60 or 65 years after a cerebral ischemic event with no findable putative cause other than the PFO [28]. As a consequence, PFO closure is exclusively recommended for such patients in all guidelines. To extrapolate indications to other embolic events in the systemic circulation, to primary prevention, or to patients who are older or have competing potential risks for systemic embolism breaches evidence-based medicine (EBM). Guidelines based on EBM so far inexcusably disregard that old and sick patients have a higher absolute risk of PFO-associated events [29]. Regardless of age and setting, it is cynical if not macabre to inform people at risk that they are to await a first event before this trivial, innocuous, and efficacious preventive procedure will be performed.

Table 2 lists reasonable indications for PFO closure. They are all supported by data [30], albeit statistically significant in randomized trials only for ischemic events [31] and migraine [6]. Yet are we and the patients not better off by extrapolating randomized data rather than actually collecting them with regard to cardiology’s simplest procedure that yields the best overall results considering positive minus negative aspects? The countless debilitating or even lethal events in control groups of future randomized trials can and should be prevented rather than just watched and marked down in study spreadsheets [29]. Most importantly, the positive aspects of any indication for PFO closure always bring about the positive effects of all other indications as collateral benefits. After a PFO closure for migraine, the patient is vaccinated permanently against paradoxical embolism into cerebral, coronary, ocular, visceral, or other systemic arteries. The other way around, a PFO can be closed after any embolic event and the patient may enjoy being cured from migraine.

**Table 2:** Proposal for PFO closure indications.

Therapeutic
- Migraine (particularly but no exclusively with aura) - Vasospastic angina pectoris - Exercise desaturation - Platypnea orthodeoxia - Sleep apnea
Primary prevention
- High-risk PFO - Large gap - Atrial septal aneurysm - Eustachian valve - Chiari network - Family history for PFO problems - Thromboembolism - Pacemaker or defibrillator electrodes/other right heart catheters - Surgery - Major abdominal or thoracic - Orthopedic - Cerebral in sitting position - Hypercoagulable blood disorders - Carcinoid tumor - Planned pregnancy
Secondary prevention
- Stroke or transient ischemic attack - Myocardial infarction - Ocular ischemia - Visceral ischemia - Peripheral ischemia - Takotsubo event - Decompression accident - High-altitude pulmonary edema or mountain sickness
Profession, hobby, or life circumstances
- Deep sea diver - Extreme mountain climber/highlander - Brass musician - Glass blower - Tile setter - Astronaut/pilot of air force jet or acrobat plane

### 4.1. Therapy

***Migraine:*** Migraine is the key indication for therapeutic PFO closure. Vasoactive triggers, foremost serotonin, are produced in the intestinal organs and become largely neutralized by the lung filter. If a gush of venous blood passes through a PFO, the systemic circulation experiences a serotonin tsunami that may activate one or several hyper-reactive receptors in the brain vessels, giving rise to a cortical spreading depression [32]. This corresponds to a transient vasoconstriction, possibly provoking an aura and being followed by reactive vasodilation causing a headache. In case of a single hypersensitive receptor, the migraine symptoms are always identical. With several hypersensitive receptors, symptoms may vary. They may still pertain to the same side in case all overreacting trigger receptors are located ipsilaterally.

PFO closure in migraine is yet another deplorable victim of EBM applied overzealously to the disadvantage of patients [29]. An elevated prevalence of PFOs among migraine patients [33] and an elevated prevalence of migraine among people with a PFO are proved and uncontested [34]. An encrusted opinion prevails, particularly but not only among neurologists, that this is explainable by common genes. This theory is refuted by the fact that PFO closure improves some migraines. Almost all comparative or randomized trials investigating PFO device closure or medical treatment alone in patients with migraine showed a numerically conspicuous improvement of migraine with PFO closure. This was more pronounced in migraineurs with aura [35]. In most non-randomized comparative reports, the difference in favour of PFO closure was statistically significant [36,37]. In all three major randomized trials, the primary endpoints were favourable for PFO closure but failed the predefined statistical significance. Most secondary endpoints showed statistically significant superiority for device closure of the PFO. High complexity of the migraine patients included, overly ambitious and ill-fated primary endpoints, and, in the studies with sham procedures, the equalizing sham (placebo) effect contributed to the missed statistical significances. Misquoting EBM, superiority trials missing their primary endpoints are repeatedly and erroneously used to bury alive a technique or a compound despite proven noninferiority and numerical superiority [29]. Neurological societies mistakably exclude PFO closure as an alternative or supplement of drug treatment for migraine, although the data leave no doubt that it will not disadvantage patients to adopt PFO closure as a therapy option for migraine. In fact, it offers them a good chance of improvement, even cure, not to mention that a mechanical vaccination as a simple and safe one-time measure is preferable to year-long medical treatment. The latter accrues cost and side effects for decades if not for life.

The maintained denial by neurologists of a merit of PFO closure in patients with migraine once again neglects that PFO closure in patients with migraine carries along for free the collateral benefit of lifelong protection against paradoxical embolism, the likes of stroke, MI, or even death. It also scotomizes the many statistically significant benefits of PFO closure in meta-analyses of randomized trials [38], inexplicably even the ones performed on individual patient data [6]. Virtually all their examined endpoints depict statistically significant advantages of PFO closure in migraine.

It pays out to search for a PFO in patients with typical migraine and close documented PFOs. At least half of such patients enjoy immediate and sustained improvement of migraine, a good percentage are even cured for good, and every single patient will be protected against paradoxical embolism through the PFO that is now closed for life.

An utterly disturbing case vignette of a 39-year-old nurse exemplifies the unfortunate prevailing attitude to the migraine and PFO issue. The patient was referred to me for refractory migraine for decades. Unfortunately, this happened more than 2 years after she had been left permanently aphasic by a stroke. A PFO was found and percutaneously occluded. The migraine vanished instantly. Had she come to my attention years earlier, she would have overcome her disabling migraine at that time. More importantly, she would not have suffered the stroke and avoided the horrid struggle to educate two teenage sons and to function socially and professionally while being unable to speak.

***Vasospastic angina pectoris:*** Like migraine, vasospastic angina can be mediated by a PFO via serotonin tsunamis and treated by PFO closure in such cases [32]. Even patients without improvement regarding vasospastic angina are mechanically vaccinated against paradoxical embolism. They lack the direct improvement but are recompensed by the collateral benefit.

***Exercise desaturation:*** Exercise desaturation occurs in some people when the PFO persistently opens during strenuous activities, causing cyanosis and limiting exercise capacity [39]. The phenomenon was documented in a third of 50 all-comers with a PFO [40]. PFO closure improved it in most patients and protected all of them against later paradoxical embolism.

***Platypnea orthodeoxia:*** The sitting position may distort the diaphragm cranially, especially in the obese elderly. This impacts the heart in a way that the PFO opens and stays patent until the body position is changed. Significant arterial desaturation ensues and is called platypnea orthodeoxia [39,41]. PFO closure solves that and does away with the accompanying high risk of paradoxical embolism of thrombi of such patients.

***Sleep apnea:*** Thera are obstructive or central forms of sleep apnea [42]. The obstructive type causes increased venous backflow into the chest during extended inspiration phases. A PFO is often opened by that. This increases the risk of paradoxical embolism and, at the same time, worsens central sleep apnea because the open PFO aggravates oxygen desaturation [43,44,45]. PFO closure improves all that.

### 4.2. Primary Prevention

It is high time to place PFO closure for primary prevention on the agenda. In a first step, it may stay limited to people who happen to be known to have a high-risk PFO.

***High-risk PFO:*** As a side note, the label ‘high-risk’ can well be employed for all PFOs presumed to have already caused an ischemic event and are considered for secondary prevention. For primary prevention, high-risk PFOs are those with high-risk anatomical and physiological features. They are large PFOs, PFOs with a spontaneous right-to-left shunt, and PFOs associated with a hypermobile septum primum, referred to as ASA [8], a Eustachian valve [9,10,11,12], or a Chiari network [13]. An ASA opens the PFO with practically every heartbeat, thereby accruing considerable overall time with a right-to-left shunt. The Eustachian valve or the Chiari network escorts the clots from the inflow of the inferior vena cava (virtually all thrombi come from there) directly through the PFO. All these anatomical variations are associated with an increased prevalence of a PFO. They are congenital and hinder PFO closure after birth.

***Family history of PFO problems:*** People with one or more close blood relatives, e.g., a twin, having suffered a PFO-mediated stroke, MI, or other ischemic accident, or having experienced improvement of migraine after PFO closure, represent additional candidates for PFO closure for primary prevention.

***Thromboembolism or hypercoagulable blood disorders:*** Patients with a history of thromboembolism or with hypercoagulability [46] should be offered primary PFO closure [47], even if they are under chronic OAC. OAC is notorious for compliance problems and there are medically imposed pauses. PFO closure knows neither compliance issues nor pauses.

***Pacemaker or defibrillator electrodes, other right heart catheters, or vena cava filter:*** All foreign bodies in the venous circulation are potential sources of thromboembolism and place PFO carriers at increased jeopardy for paradoxical embolism. This also holds true for vena cava filters. They block large clots but not the small clots typical of PFO-mediated systemic embolism. In fact, they, themselves, constitute a potential nidus for small emboli.

***Major surgery:*** Time permitting, patients undergoing major surgical interventions ought to be screened for a PFO. PFO closure should then precede surgery [48]. It reduces the risk of a stroke from 9% to 1% within the first year after surgery. Neurosurgeons operating on sitting patients in order to reduce venous bleeds depending on gravity should make sure that there is no (unclosed) PFO [49]. Brain veins that are opened during surgery have a propensity to catch air that risks bypassing the lung filter through a PFO to end up in the systemic circulation. Being lighter than blood, air will tend to climb to the brain, the topmost organ of the sitting patient.

***Carcinoid tumour:*** Left heart valve problems with carcinoid tumours are more common if the patients have a PFO [50]. These tumours typically produce serotonin in high enough doses to damage heart valves. In case the serotonin can shortcut the physiological neutralization during the lung passage, valve destruction not only pertains to the right but also to the left heart. There, it is clinically more devastating. PFO closure prevents that.

***Planned pregnancy:*** Pregnancy is associated with an increased risk of thromboembolism and a rise in right atrial pressure. This enhances the deleterious potential of a PFO. Although there are only anecdotal and no robust data to prove that the pregnancy risk be increased by the presence of a PFO [51,52], closing a PFO before a planned pregnancy cannot be wrong, considering the collateral benefits.

### 4.3. Secondary Prevention

***Stroke and transient ischemic attack (TIA):*** An embolic stroke differs from an embolic TIA only in the irreversibility of symptoms, which is subject to chance. No differences should be made in the indications for PFO screening and closure between the two. TIA symptoms may be mimicked by migraine. Especially in such cases, PFO closure, being a valuable therapy for migraine and protecting against future real embolic strokes, is the intervention of choice. The PFO cannot be acquitted in patients with a cerebral event presumed to be embolic on the basis that there are other potential stroke causes or because the patient is old or has comorbidities. The absolute yield, in contrast to the relative yield, to be expected with PFO closure, increases with the age and disease of the candidates. Compared to the old and sick, the young and so-called cryptogenic stroke patients have a lower risk of a PFO-mediated paradoxical embolism. AF is not a contra-indication to PFO closure, either. Even under OAC, PFO closure is beneficial. Paradoxical embolism is more common with AF than without, as there are more clots in the venous circulation, and it is fully preventable by PFO closure but only partially by OAC. Extended screening for occult AF in young, otherwise healthy patients with a cerebral accident makes little sense. The yield is negligible. Even if AF is detected, it is unlikely that it was responsible for the event. A high-risk PFO is a much more likely culprit. In addition, a lengthy search for AF defers or even makes the initially planned PFO closure forgotten about. Additional preventable yet un-prevented PFO-mediated events may be the price for that. Finally, an obvious and attractive approach is to simultaneously close the PFO and the LAA. This puts an end to the indication for OAC and improves longevity twofold. Both PFO closure [22] and LAA closure—compared to warfarin [53] or to novel oral anticoagulants [54]—have shown a survival benefit.

***MI:*** MI by paradoxical embolism through a PFO must be one of the most frequently misdiagnosed problems in cardiology [55]. This is particularly vexing because it is easily preventable by PFO closure. The cardiologists are the ones to blame for this oversight. In the wake of the bad example of neurologists, who, for nebulous reasons, want to have excluded all other putative causes for stroke before scouting for a PFO, cardiologists think of all imaginable causes of an MI before considering paradoxical embolism. In the absence of obvious atherosclerosis (the prime suspect), spontaneous coronary dissection, protracted spasm, or drug abuse are pondered, searched for, or incriminated right off the bat, but not a PFO. The mentioned fact goes ignored that atherosclerosis, age, and systemic disorders promote rather than exclude paradoxical embolism of a venous clot. Only a minority of interventional cardiologists list a PFO among the acknowledged causes of MI. The amount of blood supply to the coronary arteries is about 220 mL/minute at rest. This corresponds to almost a third of that to the brain (roughly 750 mL/minute). Both organs are equally jeopardized by the small clots typically passing through a PFO. Limbs and visceral organs require larger clots for serious ischemic harm. In the brain and heart, there are silent embolisms thanks to unimportant areas, pre-existing tissue scars, or collateralized territories. Accounting for all that, one should attribute to a PFO roughly one MI per three cerebral events. It has not yet been taken into account that neurology significantly underdiagnoses PFO-mediated cerebral events. Typically, they are ruled out by default in patients with the highest respective risk. The number of correctly diagnosed PFO-mediated MIs should probably be at least hundredfold higher than it currently is. Any acute MI looking even remotely embolic should prompt a search for a PFO, once the coronary situation is managed. This can be accomplished with a right heart catheterization at the end of the primary coronary percutaneous intervention. Rather than directly probing the PFO with a wire or a catheter as described above, a right atrial injection is preferable (Figure 1). There is a small but concerning risk of a thrombus saddling the PFO [24], part of which just broke off and caused the acute MI. Excluding or proving and closing a PFO in that way adds a few minutes to the coronary case and is doable day or night. It does not really increase the overall risk of the procedure but can do a lot of good. It makes sense in embolic-looking MIs irrespective of whether or not they were indeed caused by the PFO. The next MI could well be, and a mechanical vaccination against paradoxical embolism is never unwarranted. By the way, screening for a PFO with a right heart catheter is less uncomfortable for the patient than with a TEE. The features of the PFO to be respected when selecting the appropriate device size (Figure 4) can be equally well depicted in such a study with contrast medium (Figure 1). A combined coronary intervention and PFO closure is in the vetted interest of patients.

***Ocular, visceral, or peripheral ischemia, takotsubo events, decompression accidents, and high-altitude pulmonary edema or mountain sickness:*** The causative relationship of the PFO with ocular, visceral, or peripheral embolism is the same as that of cerebral and coronary ischemic events. Yet, with the exception of ocular embolism, larger clots are at stake. They are less likely to pass a regular PFO. A takotsubo event may be triggered by a PFO via a mechanism similar to the one described above for migraine or vasospastic angina pectoris. Decompression accidents and high-altitude pulmonary edema or mountain sickness in the realm of PFOs are dealt with in the paragraphs below. 

### 4.4. Profession, Hobby, and Life Circumstances

***Deep sea divers:*** Diving carries a risk for decompression accidents, mainly by nitrogen bubbles. The more prevalent ones in the venous circulation are eliminated by the lung filter. A PFO increases the risk significantly that such bubbles bypass the filter to reach sensitive organs like the brain [56]. Divers more commonly suffer cerebral events, as evidenced by lacunar brain lesions on magnetic resonance imaging, if they have a PFO [57]. PFO closure works well for preventing further decompression events [56].

***Extreme mountain climbers or highlanders:*** High-altitude cerebral and pulmonary edema, also known as acute mountain sickness, is more common in people with a PFO [58,59]. PFO closure deserves a chance to prevent or remedy that. It will improve the overall prognosis, for sure. Highlanders are known to develop chronic elevated right atrial pressures. Constriction of pulmonary arterioles occurs, secondary to low oxygen concentrations of ambient air, to slow down blood passage through the lungs for facilitating oxygen transfer to the red blood cells. Elevated right atrial pressure perpetuates a situation similar to that at the release of a Valsalva maneuver and may keep a PFO open. Highlanders with a PFO are at a substantially increased risk for paradoxical embolism. They may experience a permanent right-to-left shunt through the PFO that deteriorates the situation by further decreasing oxygen saturation in the blood and leading to additional pulmonary arterial vasoconstriction. The right atrial pressure increases further and the vicious cycle goes on.

***Brass musicians, glass blowers, or tile setters:*** Brass musicians and glass blowers use Valsalva maneuvers for work. Activities in crouching positions with a not-yet-treated PFO render one prone to oxygen desaturation and paradoxical embolism.

***Astronauts or pilots of air force jets or acrobat planes:*** High accelerations represent a risk for paradoxical embolism in PFO carriers, but only until the PFO is occluded.

## 5. Synopsis

The PFO may well top the list of underestimated, poorly worked up, and therapeutically neglected cardiovascular health hazards. Ironically, among all risk factors and medical problems in cardiology, it is the easiest to remove. PFO device closure, the most simple and innocuous procedure of interventional cardiology with likely the best overall yield, is referred to as a mechanical vaccination as it is predominantly a preventive intervention. Notwithstanding, PFO closure is therapeutically effective regarding PFO-related symptoms in a significant proportion of PFO carriers. A collateral benefit can be expected when closing a PFO to prevent a PFO-related problem, such as ischemic cerebral, coronary, ocular, visceral, or peripheral paradoxical embolism, in terms of improvement of PFO-induced migraine, exercise intolerance, etc., and vice versa. Close a PFO for one reason and you have eliminated all other PFO-related problems for free and for sure.

A PFO exists in perhaps 25% of the population, with its particularly dangerous forms in presumably 5%. Screening for and closure of the PFO as primary prevention appears advantageous and cost-effective in at least the latter group.

It is high time to list the PFO in guidelines and textbooks as a serious candidate culprit for any ischemic cerebral, coronary, or other systemic event that could be embolic, particularly in people of age or with comorbidities. The common myth that a PFO is only to be looked for in otherwise healthy and young patients with suspicion of paradoxical embolism must be laid to rest once and for all: the sicker and older the patient, the higher the prevalence of venous thrombosis and, thus, the higher the absolute probability that the PFO causes an embolic event. Along these lines, PFO-related embolic MIs are much more common than currently suspected, let alone diagnosed, especially in old and sick people. For every three emboli finding the brain, one will find a coronary artery based on the physiological systemic blood distribution.

Hardly will one ever regret having closed a PFO; much more likely will one regret not having closed a PFO.

It is not safe to await the first event before PFO closure. It may be fatal or leave behind irreversible and clinically devastating damage.

AF is not an important adverse effect of PFO closure. AF after PFO closure only occurs—in a form that is clinically relevant and needs treatment—in patients in whom AF was already imminent before. Chronic OAC that might be or become necessary because of pre-existing or new AF or a coagulation disorder provides incomplete protection against thromboembolism because of compliance problems and imposed pauses. PFO closure supplements OAC. It knows neither compliance issues nor pauses, and it causes no bleeding problems.

PFO closure will not render more intricate subsequent left atrial access for catheter-based interventions such as ablation for AF, LAA closure, or mitral valve interventions. On the contrary, a visible PFO occluder delineates, just caudal to it, a safe area for fluoroscopically guided transseptal puncture of the septum primum (Figure 3) [60].

## Figures and Tables

**Table 1:** Proposal for embolic event classification.

Arterial embolus from
- plaque - ulcer - dissection
Cardiac embolus from
- left ventricle - left atrium - left atrial appendage (typically with atrial fibrillation) - left atrial foramen pouch - myxoma or other tumour - vegetation (septic embolus)
Paradoxical embolus via
- patent foramen ovale - atrial septal defect - pulmonary arteriovenous fistula
Pulmonary venous bed embolus
Cryptogenic (or for cerebral events more appropriately ESUS *)

* ESUS = Embolic stroke of undetermined source.

## Data Availability

Not applicable.

## References

[1] Bridges N.D., Hellenbrand W., Latson L., Filiano J., Newburger J.W., Lock J.E. (1992). Transcatheter closure of patent foramen ovale after presumed paradoxical embolism. Circulation.

[2] Madhkour R., Meier B. (2018). Patent foramen ovale closure, a contemporary review. Struct. Heart.

[3] Madhkour R., Wahl A., Praz F., Meier B. (2019). Amplatzer patent foramen ovale occluder: Safety and efficacy. Expert Rev. Med. Devices.

[4] Anderson F.A., Wheeler H.B., Goldberg R.J., Hosmer D.W., Patwardhan N.A., Jovanovic B., Forcier A., Dalen J.E. (1991). A population-based perspective of the hospital incidence and case-fatality rates of deep vein thrombosis and pulmonary embolism. The Worcester DVT Study. Arch. Intern. Med..

[5] Martinez C., Cohen A.T., Bamber L., Rietbrock S. (2014). Epidemiology of first and recurrent venous thromboembolism: A population-based cohort study in patients without active cancer. Thromb. Haemost..

[6] Mojadidi M.K., Kumar P., Mahmoud A.N., Elgendy I.Y., Shapiro H., West B., Charles A.C., Mattle H.P., Sorensen S., Meier B. (2021). Pooled analysis of PFO occluder device trials in patients with PFO and migraine. J. Am. Coll. Cardiol..

[7] Meier B. (2008). Closure of the patent foramen ovale with dedicated Amplatzer occluders: Closing in on a mechanical vaccination. Catheter. Cardiovasc. Interv..

[8] Mas J.L., Saver J.L., Kasner S.E., Nelson J., Carroll J.D., Chatellier G., Derumeaux G., Furlan A.J., Herrmann H.C., Juni P. (2022). Association of Atrial Septal Aneurysm and Shunt Size with Stroke Recurrence and Benefit From Patent Foramen Ovale Closure. JAMA Neurol..

[9] Schuchlenz H.W., Saurer G., Weihs W., Rehak P. (2004). Persisting Eustachian valve in adults: Relation to patent foramen ovale and cerebrovascular events. J. Am. Soc. Echocardiogr. Off. Publ. Am. Soc. Echocardiogr..

[10] Goel S.S., Tuzcu E.M., Shishehbor M.H., de Oliveira E.I., Borek P.P., Krasuski R.A., Rodriguez L.L., Kapadia S.R. (2009). Morphology of the patent foramen ovale in asymptomatic versus symptomatic (stroke or transient ischemic attack) patients. Am. J. Cardiol..

[11] Nakayama R., Takaya Y., Akagi T., Watanabe N., Ikeda M., Nakagawa K., Toh N., Ito H. (2019). Identification of High-Risk Patent Foramen Ovale Associated with Cryptogenic Stroke: Development of a Scoring System. J. Am. Soc. Echocardiogr. Off. Publ. Am. Soc. Echocardiogr..

[12] Cai Q., Ahmad M. (2020). Eustachian valve, interatrial shunt, and paradoxical embolism. Echocardiography.

[13] Schneider B., Hofmann T., Justen M.H., Meinertz T. (1995). Chiari‘s network: Normal anatomic variant or risk factor for arterial embolic events?. J. Am. Coll. Cardiol..

[14] Gevorgyan R., Perlowski A., Shenoda M., Mojadidi M.K., Agrawal H., Tobis J.M. (2014). Sensitivity of brachial versus femoral vein injection of agitated saline to detect right-to-left shunts with Transcranial Doppler. Catheter. Cardiovasc. Interv..

[15] Tang P.T., Cahill T., Rothwell P.M., Ormerod O.J., Daniels M.J. (2020). Serial Shunt Evaluation Reveals Limitations of Contemporary Screening Studies for Patent Foramen Ovale. JACC Cardiovasc. Interv..

[16] Mojadidi M.K., Roberts S.C., Winoker J.S., Romero J., Goodman-Meza D., Gevorgyan R., Tobis J.M. (2014). Accuracy of transcranial Doppler for the diagnosis of intracardiac right-to-left shunt: A bivariate meta-analysis of prospective studies. JACC. Cardiovasc. Imaging.

[17] Karttunen V., Ventila M., Ikaheimo M., Niemela M., Hillbom M. (2001). Ear oximetry: A noninvasive method for detection of patent foramen ovale: A study comparing dye dilution method and oximetry with contrast transesophageal echocardiography. Stroke.

[18] Billinger M., Schwerzmann M., Rutishauser W., Wahl A., Windecker S., Meier B., Seiler C. (2013). Patent foramen ovale screening by ear oximetry in divers. Am. J. Cardiol..

[19] Cohnheim J. (1889). A Handbook for Practitioners and Students.

[20] Hagen P.T., Scholz D.G., Edwards W.D. (1984). Incidence and size of patent foramen ovale during the first 10 decades of life: An autopsy study of 965 normal hearts. Mayo Clin. Proc..

[21] Tanzi A., Onorato O., Casilli F., Anzola G. (2016). Is the search for right-to-left shunt still worthwhile?. Acta Neurol. Scand..

[22] Wahl A., Jüni P., Mono M.L., Kalesan B., Praz F., Geister L., Räber L., Nedeltchev K., Mattle H.P., Windecker S. (2012). Long-term propensity score-matched comparison of percutaneous closure of patent foramen ovale with medical treatment after paradoxical embolism. Circulation.

[23] Elgendy A.Y., Saver J.L., Amin Z., Boudoulas K.D., Carroll J.D., Elgendy I.Y., Grunwald I.Q., Gertz Z.M., Hijazi Z.M., Horlick E.M. (2020). Proposal for Updated Nomenclature and Classification of Potential Causative Mechanism in Patent Foramen Ovale-Associated Stroke. JAMA Neurol..

[24] Koullias G.J., Elefteriades J.A., Wu I., Jovin I., Jadbabaie F., McNamara R. (2004). Images in cardiovascular medicine. Massive paradoxical embolism: Caught in the act. Circulation.

[25] Parekh A., Jaladi R., Sharma S., Van Decker W.A., Ezekowitz M.D. (2006). Images in cardiovascular medicine. The case of a disappearing left atrial appendage thrombus: Direct visualization of left atrial thrombus migration, captured by echocardiography, in a patient with atrial fibrillation, resulting in a stroke. Circulation.

[26] Koermendy D., Nietlispach F., Shakir S., Gloekler S., Wenaweser P., Windecker S., Khattab A.A., Meier B. (2014). Amplatzer left atrial appendage occlusion through a patent foramen ovale. Catheter. Cardiovasc. Interv..

[27] Nietlispach F., Moarof I., Taramasso M., Maisano F., Meier B. (2017). Left atrial appendage occlusion. EuroIntervention.

[28] Stortecky S., da Costa B.R., Mattle H., Carroll J., Hornung M., Sievert H., Trelle S., Windecker S., Meier B., Jüni P. (2015). Percutaneous closure of patent foramen ovale in patients with cryptogenic embolism: A network meta-analysis. Eur. Heart J..

[29] Meier B., Nietlispach F. (2019). Fallacies of Evidence-Based Medicine in Cardiovascular Medicine. Am. J. Cardiol..

[30] Meier B., Nietlispach F. (2018). The evil of the patent foramen ovale: We are seeing but the tip of the iceberg. Eur. Heart J..

[31] Kent D.M., Saver J.L., Kasner S.E., Nelson J., Carroll J.D., Chatellier G., Derumeaux G., Furlan A.J., Herrmann H.C., Jüni P. (2021). Heterogeneity of treatment effects in an analysis of pooled individual patient data from randomized trials of device closure of patent foramen ovale after stroke. JAMA.

[32] Ravi D., Tobis J., Parikh R., Aboulhosn J. (2023). A New Syndrome of Patent Foramen Ovale Inducing Vasospastic Angina and Migraine. J. Am. Coll. Cardiol. Case Rep..

[33] Schwerzmann M., Nedeltchev K., Lagger F., Mattle H.P., Windecker S., Meier B., Seiler C. (2005). Prevalence and size of directly detected patent foramen ovale in migraine with aura. Neurology.

[34] Schwedt T.J., Demaerschalk B.M., Dodick D.W. (2008). Patent foramen ovale and migraine: A quantitative systematic review. Cephalalgia Int. J. Headache.

[35] Ben-Assa E., Rengifo-Moreno P., Al-Bawardy R., Kolte D., Cigarroa R., Cruz-Gonzalez I., Sakhuja R., Elmariah S., Pomerantsev E., Vaina L.M. (2020). Effect of Residual Interatrial Shunt on Migraine Burden After Transcatheter Closure of Patent Foramen Ovale. JACC Cardiovasc. Interv..

[36] Schwerzmann M., Wiher S., Nedeltchev K., Mattle H.P., Wahl A., Seiler C., Meier B., Windecker S. (2004). Percutaneous closure of patent foramen ovale reduces the frequency of migraine attacks. Neurology.

[37] Kimmelstiel C., Gange C., Thaler D. (2007). Is patent foramen ovale closure effective in reducing migraine symptoms? A controlled study. Catheter. Cardiovasc. Interv..

[38] Kheiri B., Abdalla A., Osman M., Ahmed S., Hassan M., Bachuwa G., Bhatt D.L. (2018). Percutaneous Closure of Patent Foramen Ovale in Migraine: A Meta-Analysis of Randomized Clinical Trials. JACC Cardiovasc. Interv..

[39] Mojadidi M.K., Ruiz J.C., Chertoff J., Zaman M.O., Elgendy I.Y., Mahmoud A.N., Al-Ani M., Elgendy A.Y., Patel N.K., Shantha G. (2019). Patent Foramen Ovale and Hypoxemia. Cardiol. Rev..

[40] Devendra G.P., Rane A.A., Krasuski R.A. (2012). Provoked exercise desaturation in patent foramen ovale & impact of percutaneous closure. J. Am. Coll. Cardiol. Interv..

[41] Hayek A., Rioufol G., Bochaton T., Rossi R., Mewton N., Paccalet A., Bonnefoy-Cudraz E., Thibault H., Derimay F. (2021). Prognosis After Percutaneous Foramen Ovale Closure Among Patients with Platypnea-Orthodeoxia Syndrome. J. Am. Coll. Cardiol..

[42] Oldenburg O. (2012). Cheyne-Stokes Respiration in Chronic Heart Failure, Treatment with Adaptive Servoventilation Therapy. Circ. J..

[43] Agnoletti G., Iserin L., Lafont A., Sidi D., Desnos M. (2005). Obstructive sleep apnea and patent foramen ovale: Successful treatment of symptoms by percutaneous foramen ovale closure. J. Interv. Cardiol..

[44] White J.M., Veale A.G., Ruygrok P.N. (2013). Patent foramen ovale closure in the treatment of obstructive sleep apnea. J. Invasive Cardiol..

[45] Rimoldi S.F., Ott S., Rexhaj E., de Marchi S.F., Allemann Y., Gugger M., Scherrer U., Seiler C. (2015). Patent Foramen Ovale Closure in Obstructive Sleep Apnea Improves Blood Pressure and Cardiovascular Function. Hypertension.

[46] Buber J., Guetta V., Orion D., Lubetsky A., Borik S., Vatury O., Israel A. (2021). Patent Foramen Ovale Closure among Patients with Hypercoagulable States Maintained on Antithrombotic Therapy. Cardiology.

[47] Liu K., Song B., Palacios I.F., Inglessis-Azuaje I., Deng W., McMullin D., Wang X., Lo E.H., Xu Y., Buonanno F.S. (2020). Patent Foramen Ovale Attributable Cryptogenic Embolism with Thrombophilia Has Higher Risk for Recurrence and Responds to Closure. JACC Cardiovasc. Interv..

[48] Friedrich S., Ng P.Y., Platzbecker K., Burns S.M., Banner-Goodspeed V., Weimar C., Subramaniam B., Houle T.T., Bhatt D.L., Eikermann M. (2019). Patent foramen ovale and long-term risk of ischaemic stroke after surgery. Eur. Heart J..

[49] Fathi A.R., Eshtehardi P., Meier B. (2009). Patent foramen ovale and neurosurgery in sitting position: A systematic review. Br. J. Anaesth..

[50] Douglas S., Oelofse T., Shah T., Rooney S., Arif S., Steeds R.P. (2023). Patent foramen ovale in carcinoid heart disease: The potential role for and risks of percutaneous closure prior to cardiothoracic surgery. J. Neuroendocrinol..

[51] Dark L., Loiselle A., Hatton R., Bhagwandeen R., Collins N. (2011). Stroke during pregnancy: Therapeutic options and role of percutaneous device closure. Heart Lung Circ..

[52] Bereczki D. (2016). Pregnancy and acute ischemic stroke. Orv. Hetil..

[53] Reddy V.Y., Sievert H., Halperin J., Doshi S.K., Buchbinder M., Neuzil P., Huber K., Whisenant B., Kar S., Swarup V. (2014). Percutaneous left atrial appendage closure vs warfarin for atrial fibrillation: A randomized clinical trial. JAMA.

[54] Gloekler S., Fürholz M., de Marchi S., Kleinecke C., Streit S.R., Buffle E., Fankhauser M., Haner J.D., Nietlispach F., Galea R. (2020). Left atrial appendage closure versus medical therapy in patients with atrial fibrillation: The APPLY study. EuroIntervention.

[55] Raphael C.E., Heit J.A., Reeder G.S., Bois M.C., Maleszewski J.J., Tilbury R.T., Holmes D.R. (2018). Coronary Embolus: An Underappreciated Cause of Acute Coronary Syndromes. JACC Cardiovasc. Interv..

[56] Honek J., Sramek M., Honek T., Tomek A., Sefc L., Januska J., Fiedler J., Horvath M., Novotny S., Veselka J. (2020). Patent Foramen Ovale Closure Is Effective in Divers: Long-Term Results From the DIVE-PFO Registry. J. Am. Coll. Cardiol..

[57] Billinger M., Zbinden R., Mordasini R., Windecker S., Schwerzmann M., Meier B., Seiler C. (2011). Patent foramen ovale closure in recreational divers: Effect on decompression illness and ischaemic brain lesions during long-term follow-up. Heart.

[58] West B.H., Fleming R.G., Al Hemyari B., Banankhah P., Meyer K., Rozier L.H., Murphy L.S., Coluzzi A.C., Rusheen J.L., Kumar P. (2019). Relation of Patent Foramen Ovale to Acute Mountain Sickness. Am. J. Cardiol..

[59] Allemann Y., Hutter D., Lipp E., Sartori C., Duplain H., Egli M., Cook S., Scherrer U., Seiler C. (2006). Patent foramen ovale and high-altitude pulmonary edema. JAMA.

[60] Zaker-Shahrak R., Fuhrer J., Meier B. (2008). Transseptal puncture for catheter ablation of atrial fibrillation after device closure of patent foramen ovale. Catheter. Cardiovasc. Interv..

